# Phylogenetic relationship of subterranean termite *Coptotermes gestroi* (Blattodea: Rhinotermitidae) inhabiting urban and natural habitats

**DOI:** 10.1016/j.heliyon.2023.e23692

**Published:** 2023-12-14

**Authors:** Naveeta M. Vellupillai, Abdul Hafiz Ab Majid

**Affiliations:** a Household & Structural Urban Entomology Laboratory, School of Biological Sciences, Universiti Sains Malaysia, 11800, Minden, Penang, Malaysia; b Centre for Insect Systematics (CIS), Faculty of Science and Technology, Universiti Kebangsaan Malaysia, 43600, Bangi, Selangor, Malaysia

**Keywords:** *Coptotermes gestroi*, CO1, 16S rRNA, Phylogenetics, Genetic diversity

## Abstract

*Coptotermes* termites were collected from forestry habitats at University Sains Malaysia, Penang, while urban samples were collected from residentials from Penang and Kedah, Malaysia. Mitochondrial DNA markers, Cytochrome Oxidase 1 (CO1), and 16S ribosomal RNA (16S rRNA) genes were amplified and sequenced to confirm the species of the termite samples as *Coptotermes gestroi*. Through Blastn, all 25 CO1 and 16S rRNA sequences, respectively from urban and natural habitats were found to be 99.54–100.00 % similar to *C. gestroi* reference sequences from previous studies in Peninsular Malaysia. The phylogenetic trees constructed using Neighboring-joining (NJ) and Maximum Likelihood (ML) methods resulted in CO1 sequences clustering in two clades and 16S rRNA sequences clustering in a single clade. The overall mean distance was low for the *C. gestroi* populations from natural habitats and urban settings (F_ST_ = 0.004). Analysis of natural habitat populations using CO1 sequences revealed two haplotypes within the population, with a haplotype diversity (Hd) of 0.045 ± 0.005, while the urban population shared a common haplotype with the natural habitat populations and there was no haplotype diversity recorded between the populations. Urban and natural habitats included only one haplotype for 16S rRNA sequences, indicating a lack of nucleotide diversity. Based on the findings, a non-significant difference between the natural habitat and urban population suggests *C. gestroi* inhabiting both regions likely originated from a similar source and underwent population homogeneity in different settings facilitated by anthropogenic dispersal.

## 1 Introduction

Asian Subterranean termites, *Coptotermes gestroi* (Isoptera: Rhinotermitidae), are widespread invasive pests discovered in urban environments and forest plantations across Peninsular Malaysia. Their threat is easily recognized and eliminated in populated urban areas through chemical control. Nevertheless, the recolonization and rapid proliferation of *C. gestroi* at treated sites and new grounds are presumably aided by the dispersal of the invasive termite populations from uncharted sources. Most studies focus on the prevalence of subterranean termites in urban and suburban environments. There is limited data on mature subterranean termite reservoirs in the non-agricultural ecosystems surrounding urban areas [[1], [2], [3], [4]].

Members of the genus *Coptotermes* were obligated to be subterranean termites with a large network of colonies, consisting of millions of individuals foraging at several subterranean sites [5,6]. Thus, *Coptotermes* species were traditionally classified based on their economic importance or as forest pests. In Malaysia, *Coptotermes gestroi* species were responsible for termite-induced structural damage whereas *Coptotermes kalshoveni* and *Coptotermes curvignathus* were notorious for destroying the plantation of rubber, oil palm, sugar cane, and pine wood [[7], [8], [9], [10]]. Recent reports claimed that despite the lack of concern for *C*. *gestroi* in natural habitats, the species is slowly invading peri-urban natural environments [11,12].

Studies on alate swarming patterns, foraging activities [[13], [14], [15]], and susceptibility of exotic forest tree species to Asian subterranean termites [[16], [17], [18]] were not inclusive with reliable information on the diversity of *C*. *gestroi*. Few studies involving other termite species have suggested gene flow and genetic differentiation among populations from various ecological niches [19,20].

Various studies have shed light on the phylogenetic relationship of the *Coptotermes* population throughout Malaysia. Molecular studies employing mitochondrial DNA markers (mtDNA) have determined the precise species identification and gene analysis of termites [[21], [22], [23]]. According to Cheng et al. [24], variations in the *C. gestroi* population in Peninsular Malaysia were ecotype-associated, and haplotypes were region-specific due to *C. gestroi*'s habitat preference. Subsequently, Ab Majid et al. [25] determined common haplotypes among nine Penang locales in three heavily infested urban regions. Even though phylogenetic studies have provided information on the diversity of *C. gestroi* with widespread distribution, nothing is known about the relationship between the *C. gestroi* from urban and natural habitats.

According to Evans [12], the invasive ability of *Coptotermes* sp. in natural habitats will likely be more severe as they have more food resources and attack more tree species compared to the wood species found in urban areas. As a result of human intervention, *i.e.,* transportation, deforestation, and habitat fragmentation invasive termites were observed to thrive more in urban settings, however genetic analysis by Leniaud et al. [26] evaluated unicoloniality, with low genetic divergence within the termite colonies due to relatively recent introductions and bottleneck incidence. Nevertheless, little is known about the phylogeny and genetic diversity of *C. gestroi* in natural settings. Hence, studies incorporating mitochondrial gene markers to establish the interspecific phylogeny of *C. gestroi* between natural and urban settings will significantly improve the knowledge of the diversification of the species across eco-geographic zones.

Therefore, this study aims to determine the genetic diversity and phylogenetic relationship of the *C. gestroi* population found in urban and natural habitats in Penang and Kedah. *Coptotermes* termites were collected from infested trees and tree stumps in the forestry habitats of University Sains Malaysia, Penang, while urban samples were collected from residentials from Penang and Kedah. Mitochondrial DNA markers, Cytochrome Oxidase 1 or CO1 gene, and 16S ribosomal RNA or 16S rRNA gene were used in this study for species identification, haplotype diversity, and measuring the genetic distance between termites sampled from natural and urban sites. This study also investigated the diversity of *C. gestroi* within natural and urban environments and its population connectivity.

## 2 Materials and methods

### 2.1 Field site and termite collection

Six natural habitats on Universiti Sains Malaysia, Penang's main campus and six urban residentials in Penang and Kedah. The urban sites were identified based on reported termite infestation from residential owners, whereas the natural habitats were recognized based on mud tubes on infested trees. The sampled termites were identified as *Coptotermes* spp. based on the soldiers' white waxy secretions and examination under a dissecting microscope (Leica EZ24, Leica microsystem, Singapore) of the termites' external morphology based on Tho's shape, characteristics of the head, mandibles, antenna, notum, size, and color [27]. Approximately 10 soldiers were sampled through single collection from infested residential sites. The termite soldiers were collected from sources of infestations and was stored in vials containing 70 % ethanol. As for natural habitats on Universiti Sains Malaysia campus in Penang, significant sampling with 10 soldiers collected from infested trees and Underground Monitoring Stations established surrounding the perimeter of infested trees prior to collection. Similarly, the soldier samples were stored in alcohol vials kept at −20 °C [28]. Molecular analyses were conducted on termite samples from 31 sample sites from urban and natural environments. The details of each sample's location and abbreviation are listed in Table 1, Table 2.

**Table 1:** Coding for *Coptotermes gestroi* from natural habitats in Universiti Sains Malaysia (USM), Penang. Sources: Tree and Underground Monitoring Station (UMS).

Locations	Sources	Code
BumbleDees Café, USM, Penang	Tree	BT
	UMS 1	B1
	UMS 2	B2
	UMS 3	B3
Desasiswa Restu M02, USM, Penang	Tree	ST
	UMS 1	S1
	UMS 2	S2
	UMS 3	S3
	UMS 4	S4
School of Pharmaceutical Sciences, USM, Penang	Tree	FT
	UMS 1	F1
	UMS 2	F2
	UMS 3	F3
Padang Kawad, USM, Penang	Tree	PKT
	UMS 1	PK1
	UMS 2	PK2
	UMS 3	PK3
Desasiswa Cahaya Gemilang, USM, Penang	Tree	CGT
	UMS 1	CG1
	UMS 2	CG2
	UMS 3	CG3
Kopa Arena USM, Minden, Penang	Tree	MT
	UMS 1	M1
	UMS 2	M2
	UMS 3	M3

**Table 2:** Coding for *Coptotermes gestroi* from urban sites in Penang and Kedah.

Location	Code
Teluk Kumbar, Penang	TK
Batu Maung, Penang	BM
Bertam, Penang	BR
Bandar Putra Bertam, Penang	BPB
Kulim, Kedah	KK
Bandar Baharu, Kedah	BBK

### 2.2 Genomic DNA extraction, PCR amplification, DNA sequencing and analyses

DNA was extracted from the termite soldier samples collected urban and natural sites. According to the manufacturer's instructions, the HiYield PlusTM Genomic DNA Mini Kit (Blood/Tissue/Cultured Cells) (Real Biotech Corp., Taipei, Taiwan) was used to extract genomic DNA of *C. gestroi* samples [29]. The extraction process involves the crushing of the head capsule of a termite soldier samples, incubation of specimen in lysis buffer and Proteinase K, centrifugation after application of two types of washing buffer and elution step performed thrice to achieve 100 μL of genomic DNA.

PCR amplification was attempted using two mitochondrial DNA markers, CO1 and 16S rRNA genes to determine the species of termites. The CO1 gene was amplified using custom primers, TLF (5′-TTCGGAGCTTGATCAGGTATGGTA-3′) and TLR (5′-TATAGA TAGTACGTAGTGGAA-3′) as described by Aanen et al. [30], whereas 16S rRNA gene was amplified using primers 16Sar (5′-CGCCTGTTTATCAAAAACAT-3′) and 16Sbr (5′CCGGTCTGAACTCAGATCACGT-3′) which were adapted from Palumbi [31]. The PCR was amplified using a thermocycler machine (TaKaRa PCR Thermal Cycler Dice mini, Takara Bio Inc., Japan). The PCR reaction mixture contained 25 μL Master mix buffer (EconoTaq, Lucigen, USA), 5 μL of DNA, 1 μL (10 μM) of each primer and distilled water was added up to 50 μL of PCR reaction volume. The PCR reaction profile consists of 36 cycles of denaturation at 94 °C for 30 s, annealing at 48 °C and 47 °C for the CO1 and 16S rRNA primer, respectively, extension at 72 °C for 1 min, and final extension at 72 °C for 10 min. The PCR products were visualized by electrophoresis on 1.0 % Agarose gel. Purification and sequencing for the PCR products were conducted by service provider, First Base Laboratories Sdn. Bhd., Malaysia.

### 2.3 Phylogenetic analysis and genetic variation of CO1 and 16S rRNA sequences

The CO1 and 16S rRNA sequences were checked for quality using Bioedit (v7.2), and the sequences were compared to the NCBI database. The sequences that corresponded most closely to the sequences in GenBank were assigned with accession numbers. Clustal W in Molecular Evolutionary Genetic analysis (MEGA11) software [32] was used to align sample sequences from urban, wild, and outgroup settings. Table 3 lists the accession numbers of the sample sequence and outgroups. Neighboring-joining (NJ) and Maximum Likelihood (ML) methods were utilized to create phylogenetic trees. The stability and reliability of phylogenetics trees were evaluated using a bootstrap value of 1000 resampling. DNA Sequence Polymorphism (DNAsp) (v6. 12.03) was used to estimate the pairwise genetic distance (F_ST_) between populations of urban and natural habitats, as well as to determine the haplotype variation frequencies, nucleotide diversity and segregating sites for CO1 and 16S rRNA sequences of termites collected from urban and natural habitats.

**Table 3:** Termite sequences used in the phylogenetic and population genetic analyses.

Species	Code	State/Country	Reference	GenBank accession number	
				CO1	16S
*C. gestroi*	BT	Penang	Present study	OL881240	OL966565
*C. gestroi*	B1	Penang	Present study	OL881241	OL966568
*C. gestroi*	B2	Penang	Present study	OL881242	OL966567
*C. gestroi*	B3	Penang	Present study	OL881243	OL966566
*C. gestroi*	ST	Penang	Present study	OL881264	OL966544
*C. gestroi*	S1	Penang	Present study	OL881260	OL966548
*C. gestroi*	S2	Penang	Present study	OL881261	OL966547
*C. gestroi*	S3	Penang	Present study	OL881262	OL966546
*C. gestroi*	S4	Penang	Present study	OL881263	OL966545
*C. gestroi*	FT	Penang	Present study	OL881248	OL966557
*C. gestroi*	F1	Penang	Present study	OL881249	OL966560
*C. gestroi*	F2	Penang	Present study	OL881250	OL966559
*C. gestroi*	F3	Penang	Present study	OL881251	OL966558
*C. gestroi*	PKT	Penang	Present study	OL881259	OL966549
*C. gestroi*	PK1	Penang	Present study	OL881256	OL966552
*C. gestroi*	PK2	Penang	Present study	OL881257	OL966551
*C. gestroi*	PK3	Penang	Present study	OL881258	OL966550
*C. gestroi*	CGT	Penang	Present study	OL881247	OL966561
*C. gestroi*	CG1	Penang	Present study	OL881244	OL966564
*C. gestroi*	CG2	Penang	Present study	OL881245	OL966563
*C. gestroi*	CG3	Penang	Present study	OL881246	OL966562
*C. gestroi*	MT	Penang	Present study	OL881255	OL966553
*C. gestroi*	M1	Penang	Present study	OL881252	OL966556
*C. gestroi*	M2	Penang	Present study	OL881253	OL966555
*C. gestroi*	M3	Penang	Present study	OL881254	OL966554
*C. gestroi*	TK	Penang	Present study	OP422952	OP425197
*C. gestroi*	BM	Penang	Present study	OP422956	OP425201
*C. gestroi*	BR	Penang	Present study	OP422954	OP425199
*C. gestroi*	BPB	Penang	Present study	OP422955	OP425200
*C. gestroi*	KK	Kedah	Present study	OP422953	OP425198

## 3 Results

### 3.1 Phylogenetic analysis of CO1 and 16S rRNA sequences

**Fig. 1:**
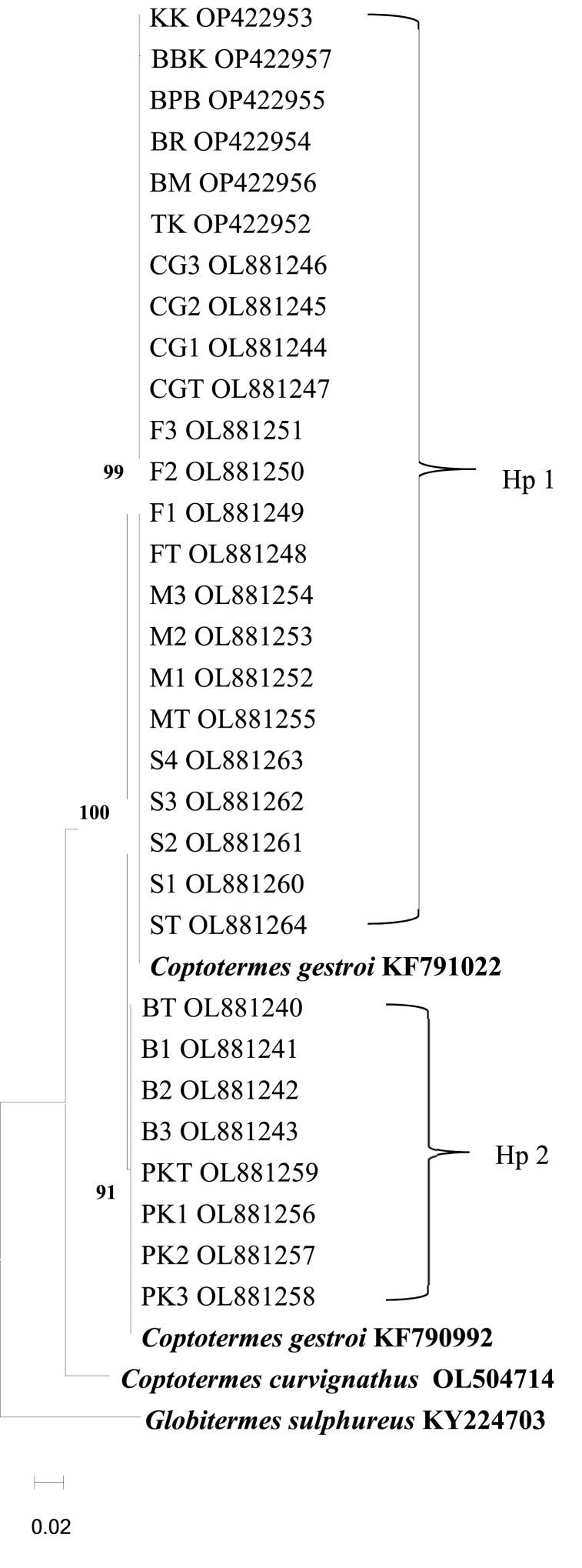
Neighboring-joining phylogenetic tree based on CO1 sequences. *Coptotermes curvignatus* OL504714 and *Globitermes sulphureus* KY224703 are the outgroups. Two haplotypes (Hp 1 and Hp 2) are detected. Abbreviation refers to Table 1, Table 2, Table 3.

**Fig. 2:**
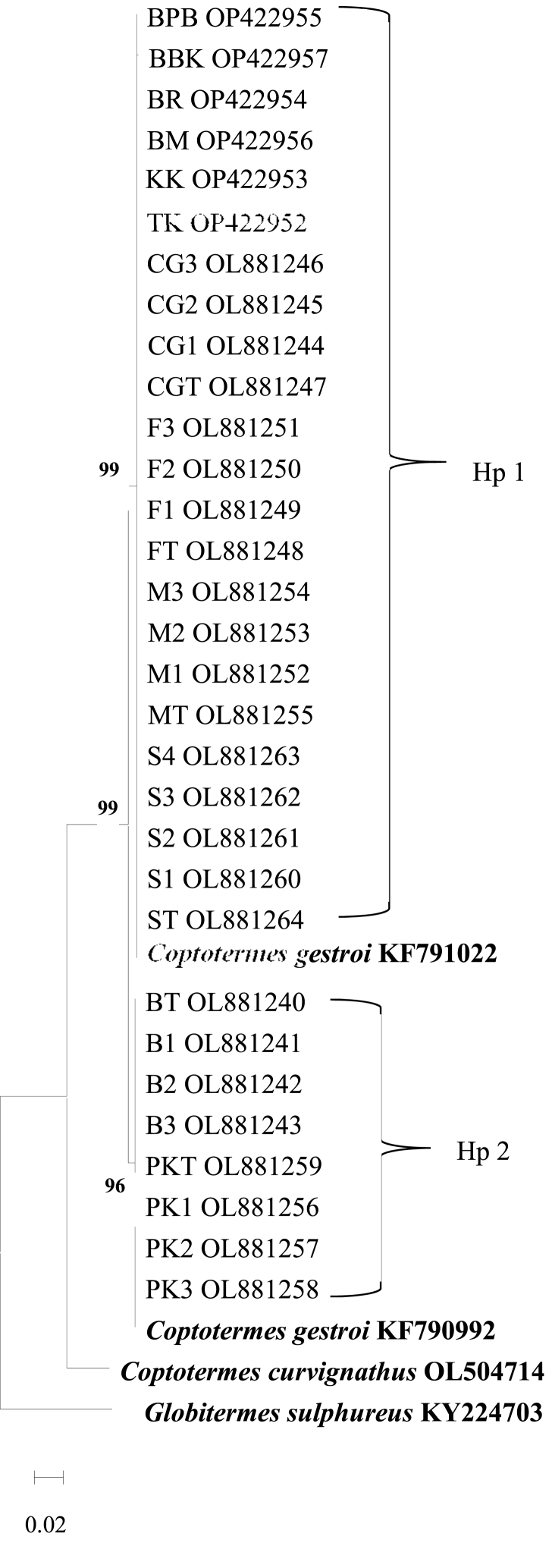
Maximum-likelihood phylogenetic tree based on CO1 sequences. *Coptotermes curvignatus* OL504714 and *Globitermes sulphureus* KY224703 are the outgroups. Two haplotypes (Hp 1 and Hp 2) are detected. Abbreviation refers to Table 1, Table 2, Table 3.

All CO1 sequences of urban and natural habitat samples from Penang and Kedah were confirmed 99.54–100.00 % similar to *C. gestroi* (KF790992 and KF791022) specimens from Peninsular Malaysia. The COI gene sequences had an overall of 506 base pairs. Fig. 1, Fig. 2 depict the two phylogenetic trees constructed for CO1 sequences using the NJ method with p-distance and the ML method with the Hasegawa-Kishino-Yano model (HKY). All the sequences from the natural habitats were clustered into two clades, and urban residential CO1 sequences shared a clade with natural habitat sequences.

**Fig. 3:**
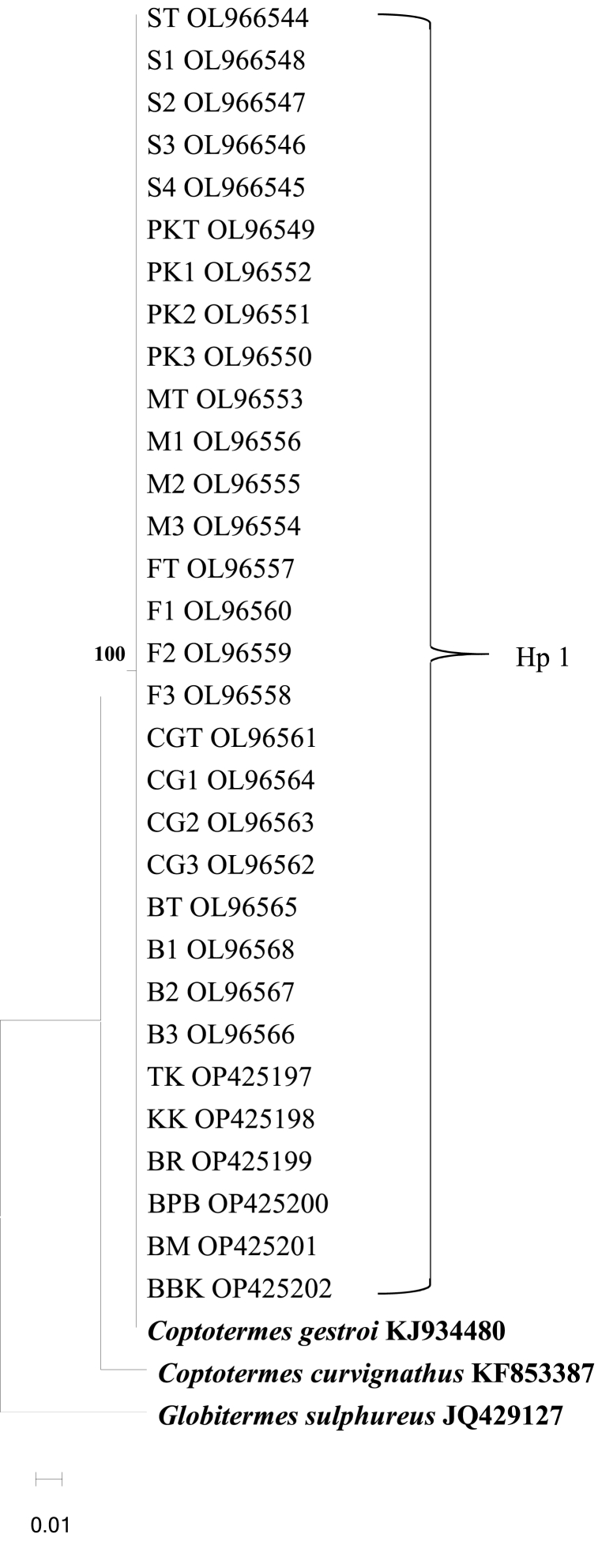
Neighboring-joining phylogenetic tree based on 16S rRNA sequences. *Coptotermes curvignatus* KF853387 and *Globitermes sulphureus* JQ429127 are the outgroups. A single haplotype (Hp 1), was detected. Abbreviation refers to Table 1, Table 2, Table 3.

**Fig. 4:**
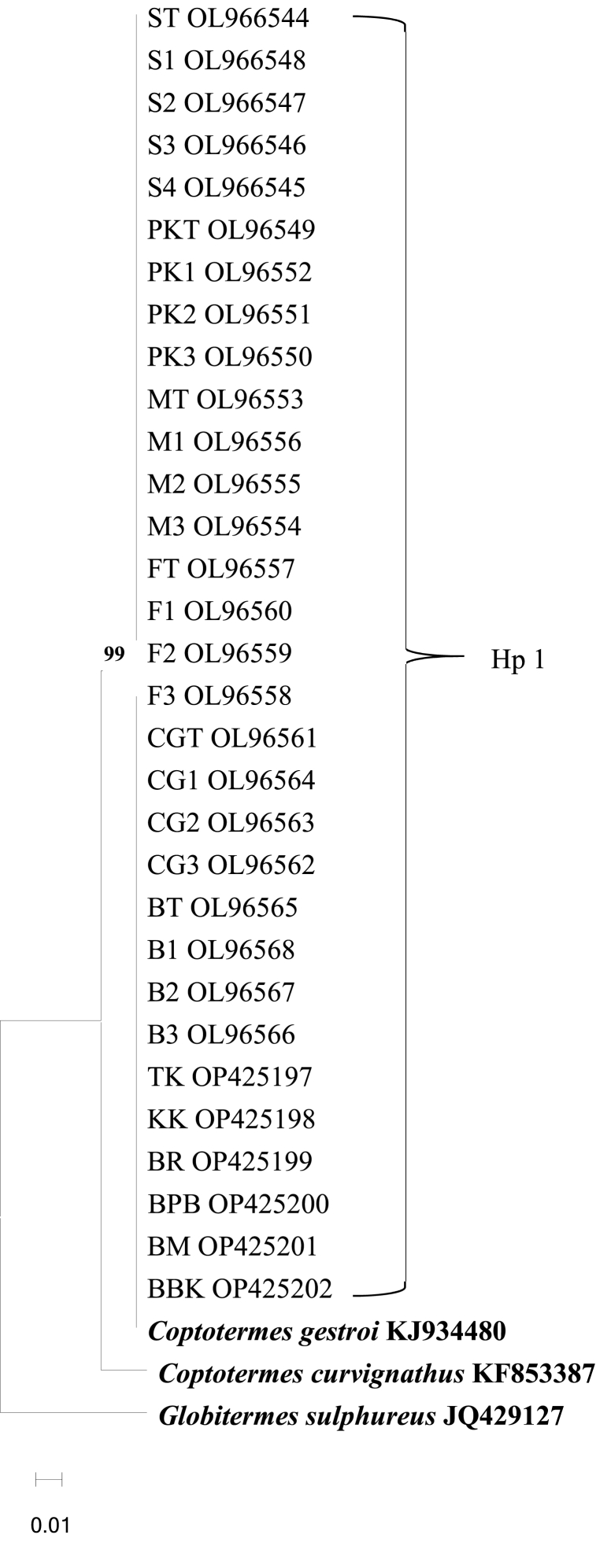
Maximum-likelihood phylogenetic based on 16S rRNA sequences. *Coptotermes curvignatus* KF853387 and *Globitermes sulphureus* JQ429127 are the outgroups. A single haplotype (Hp 1), was detected. Abbreviation refers to Table 1, Table 2, Table 3.

For 16S rRNA gene, it was confirmed the sequence of all termite samples were similar to *C. gestroi* (KF790971) specimen from Peninsular Malaysia by 100.00 % using Blastn. The 16S rRNA gene sequences had an overall of 493 base pairs. Fig. 3, Fig. 4 depict the phylogenetic trees generated using NJ method and ML method with Hasegawa-Kishino-Yano model (HKY) are shown in. All 16S rRNA sequences from urban and natural habitat from Penang and Kedah were clustered into one clade.

### 3.2 Pairwise diversity

Table 4 depicts intra-population and inter-population differentiation of *C. gestroi* from the natural habitats at Universiti Sains Malaysia, Penang, and urban residentials in Penang and Kedah are shown in. The was no genetic diversity between populations in natural habitat. The pairwise genetic distance (F_ST_) value between natural habitat and urban populations was equally insignificant. The overall mean distance was 0.003 for urban and 0.001 for natural populations, while the mean distance between the two regions was 0.004.

**Table 4:** Pairwise distance comparisons of CO1 sequences of *C. gestroi* from natural habitat and urban population of Penang and Kedah.

Population	Overall mean distance ±S.E.	F_st_	df	Chi^2^	P-value
**Urban**	0.003 ± 0.001	–	–	–	–
**Urban *vs.* Natural habitat**	0.004 ± 0.001	0.000	4	2.962	0.5642 (ns)
**Natural habitat**	0.001 ± 0.001	–	–	–	–

### 3.3 Genetic diversity of CO1 and 16S rRNA sequences

CO1 sequences from natural habitats were expressed in two haplotypes. The overall haplotype diversity (Hd) was 0.045 ± 0.005, while nucleotide diversity was 0.001 (Table 5). As for CO1 sequences from urban residentials a common haplotype was detected with no divergence of haplotype and nucleotides. The *C. gestroi* samples were collected from urban settings of the state of Penang and Kedah and samples from four natural habitat sites of Universiti Sains Malaysia, Penang: 1) Desasiswa Restu M02, 2) School of Pharmaceutical Sciences, 3) Kopa Arena USM, Minden, and 4) Desasiswa Cahaya Gemilang shared a single haplotype. While *C. gestroi* from the natural habitats of Universiti Sains Malaysia, Penang: BumbleDees Café and Padang Kawad were segregated with a different haplotype. Overall, the CO1 sequences of natural habitat samples were determined with two haplotypes and urban sample CO1 sequences shared a common haplotype with natural habitat *C. gestroi* samples. There was also no haplotype and nucleotide diversity for all 16S rRNA sequences of *C. gestroi* from both natural and urban sites.

**Table 5:** Genetic diversity of *C. gestroi* from natural habitat population in Universiti Sains Malaysia, Penang.

Population	Natural habitats of USM, Penang
**n**	25
**No. haplotypes**	2 (P = 0.987)
**Haplotype diversity (Hd) ± SD**	0.045 ± 0.005 (P = 0.843)
**Nucleotide diversity (Pi)**	0.001
**No. segregating sites**	2 (P = 0.980)
**Theta-K**	0.045 (P = 0.842)
**Theta-W**	0.265 (P = 0.980)

P > 0.01 indicated no significant difference.

## 4 Discussion

Phylogenetic analysis of CO1 and 16S rRNA sequences verified that termites collected from natural and urban environments were *Coptotermes gestroi*, commonly found in the Peninsula. All CO1 sequences were clustered with *C. gestroi* reference sequences from GenBank into two clades supported by a high bootstrap value and closely related species, *C. curvignatus* and *G. sulphureus,* were separated into distinct branches (Fig. 1, Fig. 2). 16S rRNA sequences from urban and natural sites were grouped in one clade based on *C. gestroi* reference sequences (Fig. 3, Fig. 4). The results depicted that CO1 and 16S rRNA markers were sufficient for *C. gestroi* molecular identification. In accordance with a review by De Mandal et al. [33], both CO1 and 16S rRNA markers have reportedly provided robust phylogenetic analyses, species identifications and revealed the evolutionary relationship of termite genera.

The inconsistent phylogenetic tree pattern between CO1 and 16S rRNA gene sequences revealed genetic variation exhibited by the fast-evolving CO1 gene compared to 16S rRNA gene [34]. The CO1 sequences formed two clades phylogenetic trees that supported the haplotype diversity between *C. gestroi* populations (Fig. 1, Fig. 2), while the 16S rRNA sequences generated phylogenetic trees with only one major haplogroup (Fig. 3, Fig. 4). The CO1 gene evolves under the selective constraints of synonymous substitutions, but the 16S rRNA gene is highly conserved and accumulates compensating mutation. These are the most likely causes for the variation in pattern between the two indicators [[35], [36], [37]].

The data identified a low level of haplotype diversity for natural habitat population. Moreover, the haplotype groups observed in the natural habitat population were common *C. gestroi* haplotypes found in the West coast region of Peninsular Malaysia, and these haplotypes were associated to ancestral sequences of *C. gestroi* colonies located in a wide range of habitats such as urban, semi-urban, plantations, and forests. Therefore, the haplotypes of populations inhabiting natural habitat derived from closely related ancestral states coexisted in the same region. The results were consistent with reports from previous studies on *Coptotermes* spp. genetic divergence in Malaysia [38,39].

In addition, the urban population of Penang and Kedah lacked a unique polymorphic haplotype and shared a haplotype with the natural habitat population from Universiti Sains Malaysia, Penang. The absence of haplotype diversity in urban and natural populations may suggest *C. gestroi* population inhabiting both regions likely originated from a single ancestor as predicted by coalescence theory [[40], [41], [42]]. Even though the mechanism of dispersal of common haplotype between urban and natural region remains unclear, the information proposes the probability of *C. gestroi*'s invasion in both regions and the possibility of natural colonies becoming urban exploiters and vice versa [43,44].

Besides, genetic heterogeneity for *C. gestroi* population is likely more in densely urbanized sites than in undisturbed natural habitats. However, based on the results from this study, the population diversity of *C. gestroi* was unaffected by the modifications caused by urbanization. Intriguingly, the urban *C. gestroi* population are scarce in diversity. These urban populations probably likely expanded recently and experienced bottleneck effects attributable to effective pest control management, resulting in reduced population size and some founder effect [45,46].

No significant difference was found (Table 4) when examining the population's isolation by distance from natural habitats and urban residentials. Yeap et al. [39] demonstrated similar outcome showing low pairwise genetic differentiation (F_ST_ = 0.073) among the Peninsular Malaysia *C. gestroi* population. In contrast, significant pairwise distance values were also estimated between termite subpopulations if dispersal is obstructed by geographical isolation resulting in limited gene flow [[47], [48], [49]]. Hence the results showed no discrepancy in genetic variation in population from urban and natural habitat, despite their distinct geographical settings.

The fact that the *C. gestroi* samples collected within natural habitats of Universiti Sains Penang and urban *C. gestroi* samples from Kedah and Penang share a single haplotype proposes the species support native and introduced status in parts of the states. However, it is unknown on the origin of the species from urban and natural habitats. The current study suggests the *C. gestroi* population in urban environments likely originated from populations in natural habitats as the haplotype was more abundant in natural habitat sites. The dispersal of the introduced colonies is probably facilitated by human-mediated transportation as the *C. gestroi* population in natural habitat is situated on an island hence the physical barrier between urban settings may prevent natural migration [50,51]. The variability of haplotypes within natural habitats can be explained via colony-level diversity, but the genetic analysis using mitochhondrial DNA markers are unable to detect the *C. gestroi* soldier samples belonging to different colony. As most of the *C. gestroi* colonies have an intrinsic breeding system there were probably some individuals from extended families headed by multiple neotenics and mixed family colonies among the natural habitat *C. gestroi* population causing the difference in haplotypes [52].

## 5 Conclusion

This study examines the phylogeny and diversity of *C. gestroi* from contrastingly different environments (urban and natural). The species identification using mitochondrial markers, CO1 and 16S rRNA confirmed the samples were *C. gestroi* and phylogenetic analysis verified the samples were related to reference sequences of *C. gestroi* from earlier studies found in the Peninsula Malaysia. The mitochondrial markers CO1 gene resolved two haplotypes for the population found in the natural habitats of Universiti Sains Malaysia, Penang, while the urban population from Penang and Kedah shared a common haplotype with the natural habitat population. The 16S rRNA gene resolved a single haplotype for both urban and natural habitat populations. Based on the pairwise distance analysis and genetic diversity analysis, a non-significant divergence between natural habitat and urban population suggests population homogeneity. Low levels of haplotype diversity and lack of heterogeneity between urban and natural habitat populations suggest that *C. gestroi* populations likely originated from the same source with dispersal facilitated by human-mediated transportation. Further research on the population structure of *C. gestroi* with other highly polymorphic markers, such as microsatellite markers will be efficient in order to explain the nature of haplotype variability in natural habitats and the lack of genetic differences between urban and natural *C. gestroi* populations.

## Authors contribution statement

All authors listed have significantly contributed to the development and the writing of this article.

## Data availability

The data generated and analyzed during the current study are not deposited in publicly available repository and the data will be made available upon request.

## Fundings

The research was supported under Research University Grant (Rui) 1001/PBIOLOGI/8011104.

## CRediT authorship contribution statement

**Naveeta M. Vellupillai:** Writing – review & editing, Writing – original draft, Investigation, Formal analysis, Data curation. **Abdul Hafiz Ab Majid:** Writing – review & editing, Writing – original draft, Validation, Supervision, Software, Resources, Project administration, Methodology, Investigation, Funding acquisition, Conceptualization.

## Declaration of competing interest

The authors declare that they have no known competing financial interests or personal relationships that could have appeared to influence the work reported in this paper.
